# The diagnostic value of Superb Microvascular Imaging in identifying benign tumors of parotid gland

**DOI:** 10.1186/s12880-020-00506-y

**Published:** 2020-09-16

**Authors:** Lihui Zhao, Yiran Mao, Jie Mu, Jing Zhao, Fangxuan Li, Sheng Zhang, Xiaojie Xin

**Affiliations:** 1 Department of Ultrasound, Tianjin Medical University Cancer Institute and Hospital; National Clinical Research Center for Cancer, China; Key Laboratory of Cancer Prevention and Therapy, Tianjin;Tianjin’s Clinical Research Center for Cancer, Binshuixi Road, Hexi District, Tianjin, 300060 China; 2 Department of Cancer Prevention, Tianjin Medical University Cancer Institute and Hospital, Tianjin, China

**Keywords:** Superb microvascular imaging, Parotid gland tumors, Pleomorphic adenoma, Warthin’s tumor, Basal cell adenoma

## Abstract

**Background:**

We compared the ultrasound features, superb microvascular imaging (SMI) and micro vessel density (MVD) of pleomorphic adenoma (PA), Warthin’s tumor (WT) and basal cell adenoma (BCA) to explore the clinic value of SMI in differential diagnosis of benign tumors of parotid gland.

**Methods:**

The vascular distributions and grade by color doppler flow imaging (CDFI) and SMI, as well as vascular index (VI) of 249 parotid gland masses from 217 patients were analyzed.

**Results:**

The internal echogenicity of BCA are more homogeneous in comparing with WT and PA(*P* < 0.05). By SMI, the vascular distribution and vascular grade in PA were mainly peripheral (33.1%) and avascular (25.7%), Grade 1 (27.8%) and Grade 0 (25.7%). WT were mainly central (31.3%) and mixed distribution (34.9%), in Grade 3 (37.3%) and Grade 2 (36.2%). BCA was mainly peripheral (33.3%) and mixed distribution (33.3%), in Grade 2 (33.3%) and Grade 3 (33.3%). The overall detection rate of SMI for vascular Grade 2 and 3 was significantly higher than that of CDFI (*P* < 0.05). Both VI and MVD were lowest in PA, highest in WT (*P* < 0.001). The VI by SMI was correlated with MVD (*P* < 0.001). The correlation index between vascular distribution and grade by SMI and MVD were significantly higher than CDFI.

**Conclusion:**

SMI can provide low-velocity blood flow information, which is helpful for the differential diagnosis of common benign tumors of parotid gland, and is expected to be more widely used.

## Background

Parotid gland tumors account for 3–6% of head and neck tumors [1]. 80% parotid gland tumors are benign tumors, in which pleomorphic adenoma (PA), Warthin’s tumor (WT) and basal cell adenoma (BCA) account for the top three [2]. Different types of parotid gland tumors have different biological behaviors; therefore, different types of parotid gland tumors have different operation principle. PA has complex components, often presents multicenter growth, tends to infiltrate into the surrounding tissues and may recur after resection. Additionally, 2–25% PA has malignant tendency. In order to ensure the complete resection of tumors, reserve normal tissues maximumly and reduce recurrence and postoperative complications, tumor resection including superficial parotid gland resection is recommended for PA. WT is often bilateral and multifocal and has relatively simple components and less postoperative recurrence. Thus, exenteration of the tumor is recommended. Some BCAs can grow in a multicentric and multifocal manner, and 4% BCAs have malignant tendency. Thus, all involved tissues need to be completely removed during the operation for BCA. Although basal cell adenoma was first reported by Kleinsasser and Klein in 1967, until 1991 it was first time that the WHO classified basal cell adenoma (BCA) as a independent solid tumor type. There are many studies on parotid PA and WT, but few reports on BCA. Actually, BCA is not rare in clinic, while the misdiagnosis rate for BCA is as high as 50% [3].

Preoperative differential diagnosis is important, particularly for surgical decision making. However, there are many ultrasonic similarities between these three types of parotid gland tumors [3, 4]. Although high-frequency color doppler ultrasonography has become the preferred method of parotid gland imaging examination and has certain value in providing tumor location diagnosis and blood flow information, it has limited ability in the differential diagnosis of parotid gland masses. Contrast-enhanced ultrasound (CEU) can provide the perfusion of micro vessels in the tumor, and the characteristic curve of CEU can be used to analyze relevant parameters to improve the diagnostic accuracy of the tumor [5–7]. However, CEU is expensive and time-consuming. Superb microvascular imaging (SMI), as a new ultrasound technology, is able to use adaptive algorithm to separate low-speed blood flow signals from slow mixed clutter signals, so as to display the micro-low-speed blood flow signals clearly and completely. Recent studies show that, compared with color doppler flow imaging (CDFI). It can display the low-velocity blood flow in breast and kidney tumors more sensitively [8–10]. It is saving time and no extra cost, as well as can carry out true and meticulous microangiography of the lesion site without contrast enhancement.

Micro vessel density (MVD) is an important indicator reflecting tumor angiogenesis [11], which was proposed by Weidner in 1991. It is also a quantitative analysis method of tumor angiogenesis capacity. So MVD is widely used in most angiogenesis studies. This method is based on the immunohistochemical analysis of postoperative pathological tissue paraffin section specimens. The number of micro vessels under high magnification is the gold standard for the evaluation of tumor micro vessels. CD34 antigen had highest specificity for vascular endothelial cells. Therefore, at present, most studies on tumor angiogenesis use CD34 to label vascular endothelial cells and calculate the MVD value of microvascular density.

At present, there are few clinical studies on ultra-micro angiography of parotid gland tumors [12]. In this study, we compared the diagnostic performance of SMI and CDFI, and analysis correlation between SMI, CDFI and MVD, so as to explore the clinic value for SMI assess micro vascular density.

## Methods

### Patients

This study was approved by the ethics committee of Tianjin Medical University Cancer Institute and Hospital. Written consents were obtained from each patient.

We collected the clinicopathological and ultrasonic data of patients who underwent surgical treatment for parotid gland neoplasm in Tianjin medical university cancer institute and hospital between Dec, 2015 and Feb, 2019.

Inclusion criteria: 1. Perioperative ultrasound examine found focal parotid gland solid or cystic lesion; 2. Postoperative pathological examination confirmed PA of parotid gland, BCA or WT; 3. Preoperative ultrasound SMI examination was performed; 4. No other treatment was performed for parotid lesions; 5. No previous parotid gland surgery.

Exclusion criteria: 1. Patients with parotid mass without surgical treatment; 2. Other rare benign tumors of parotid gland; 3. Parotid granulomatous inflammatory lesions; 4. Enlarged lymph nodes in parotid gland; 5. Parotid carcinoma and metastatic lesions;

### Equipment and ultrasound examination

Sonography was performed with Aplio 500 ultrasound machines (Toshiba Medical Systems, Tokyo, Japan) equipped with high frequency linear array probe PLT-805AT with a central frequency of 7.0 ~ 12.0 MHz. All ultrasound images were evaluated and recorded by two experienced sonologists. Interclass correlation coefficient (ICC) values for all conventional ultrasound features, CDFI and SMI were 0.873–0.955. All sonologists had at least 8-years experiences for ultrasonic diagnosis, received professional training of SMI and applied it at least for 3 years.

All patients underwent ultrasound examination within 3 days before surgery, including two-dimensional ultrasound, color doppler, and SMI images. SMI image includes grayscale mode (mSMI) and color mode (color, cSMI).

The patient is in supine position and fully expose the parotid gland area. The location, size, boundary, shape, internal echogenicity and other characteristics of the tumor were determined by continuous scanning with conventional ultrasound. CDFI, cSMI and mSMI were activated to observe the blood flow of the tumor, and the color gain was adjusted to avoid all false images and just show the small blood vessels clearly. Based on our previous experience, we found that when the CG was 40, the blood flow display in most parotid masses was relatively clear. In order to ensure the consistency of the experimental conditions, we uniformly set the CG at 40. The speed scale of SMI was 0.8–1.2 cm/s. The sampling frame contained the tumor and its surrounding area of about 1 cm. During scanning, the tumor should not be pressurized, and the patient should be instructed to breathe calmly and avoid swallowing.

On the basis of two-dimensional localization of the tumor, the SMI software was opened to scan the parotid gland tumor from multiple angles and planes. The largest diameter plane of the tumor and the section with the most abundant blood flow were observed and analyzed, and the images were stored. The vascular distribution type and vascular grade of the parotid gland tumor detected under the cSMI and mSMI modes were recorded respectively. In the cSMI model, parotid tumor was taken as region of interest (ROI), the margin of ROI area was manually traced in triple, and vascular index (VI) in ROI area was calculated automatically by the system. Record relevant data and images, the mean value of VI were applied.

Vascular Distributions including Avascular, Central, Peripheral, Mixed [13]. Conforming to Adler’s method, the vascularity was subjectively determined as absent (Grade 0), Grade 1, minimal blood flow, 1 ~ 2 punctate or fine rod vessels in the lesion; grade 2, moderate blood flow, 3 ~ 4 punctate vessels or 1 important vessel can be seen (the length can be close to or exceed the radius of the lesion); Grade 3, rich blood flow, more than 5 punctate vessels or 2 longer vessels were observed.

**Fig. 1:**
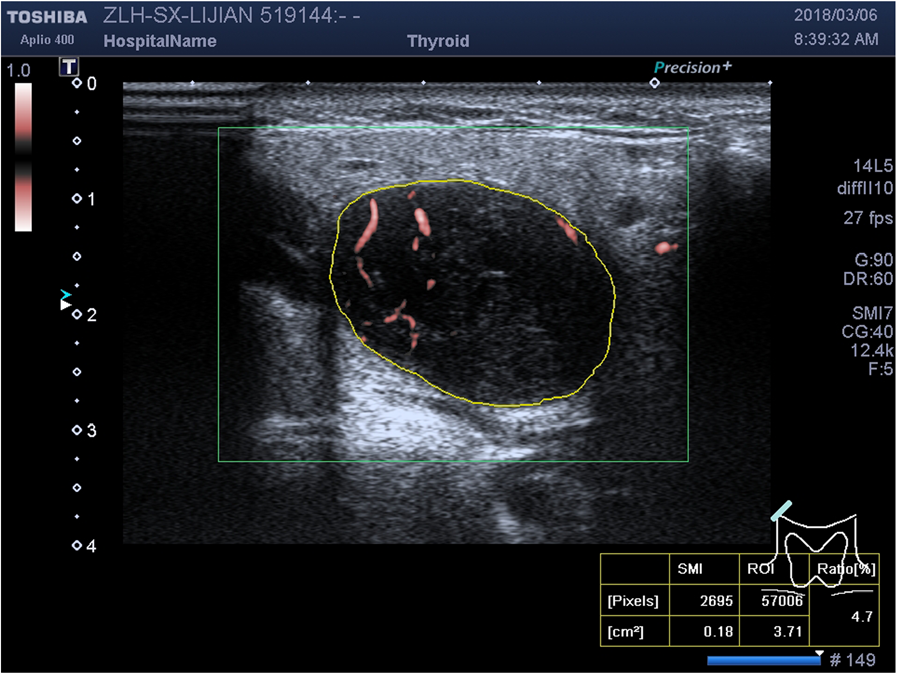
Trace the ROI and calculate VI by cSMI of a WT case

Vascular index (VI): shown in Fig. 1, after cSMI images were taken, manually trace the boundary of the tumor, and the recorded the whole tumor was regarded as the ROI, and the system automatically calculated the VI with in the described area. VI refers to the ratio between the pixel of doppler signal and the pixel of the whole lesion. Each tumor was recorded 3 times and averaged.

### Microvascular density (MVD)

Immunohistochemical (IHC) for MVD of parotid gland mass was routinely labeled by mouse anti-human CD34 monoclonal antibody. The highest density of micro vessels in the tumor tissue was found in the low-power microscope field according to the Weidner method [14], and then the number of micro vessels in the three fields was randomly observed under the high-power microscope field, and the average value was taken as the MVD value of this case group.

Micro vessel count: whether lumen or not, the single endothelial cell or group of endothelial cells stained brown and yellow were counted as 1 blood vessel. If it is suspected to be the same blood vessel or branch of blood vessel, further careful identification, as long as they are not connected to each other, count respectively. The muscular layer is thick and the lumen red blood cell count is > 8 blood vessels are not counted. Two experienced pathologists count micro vessels in double-blind.

### Statistical analysis

SPSS 23.0 software was used for statistical analysis (IBM, Corporation, Armonk, NY). Mean ± standard deviations adopted for describe the continues data, and one-way ANOVA was applied for comparing continues data. Chi-square test was applied to the categorical variables. Interclass correlation coefficient (ICC) was used to assess the repeatability of parotid mass analysis by two sonographers. Pearson and Spearman correlation analysis was used to analyze the correlation between vascular index VI and MVD in parotid gland tumors. All the statistical tests were bilateral, *P* < 0.05 was statistically significance.

## Results

### Patients

A total of 217 patients enrolled in this study, 104 were males and 113 were females, the age range was between 7 ~ 77 years, with an average age of 50.17 ± 14.96 years. There were 249 parotid gland masses in this study, with 185 cases of solitary tumor and 32 cases of multiple tumor. The size of the masses was 0.8 ~ 8.2 cm, with an average size of 2.91 ± 1.08 cm.

According to postoperative pathological types, patients were divided into three groups:

#### PA group

**Fig. 2:**
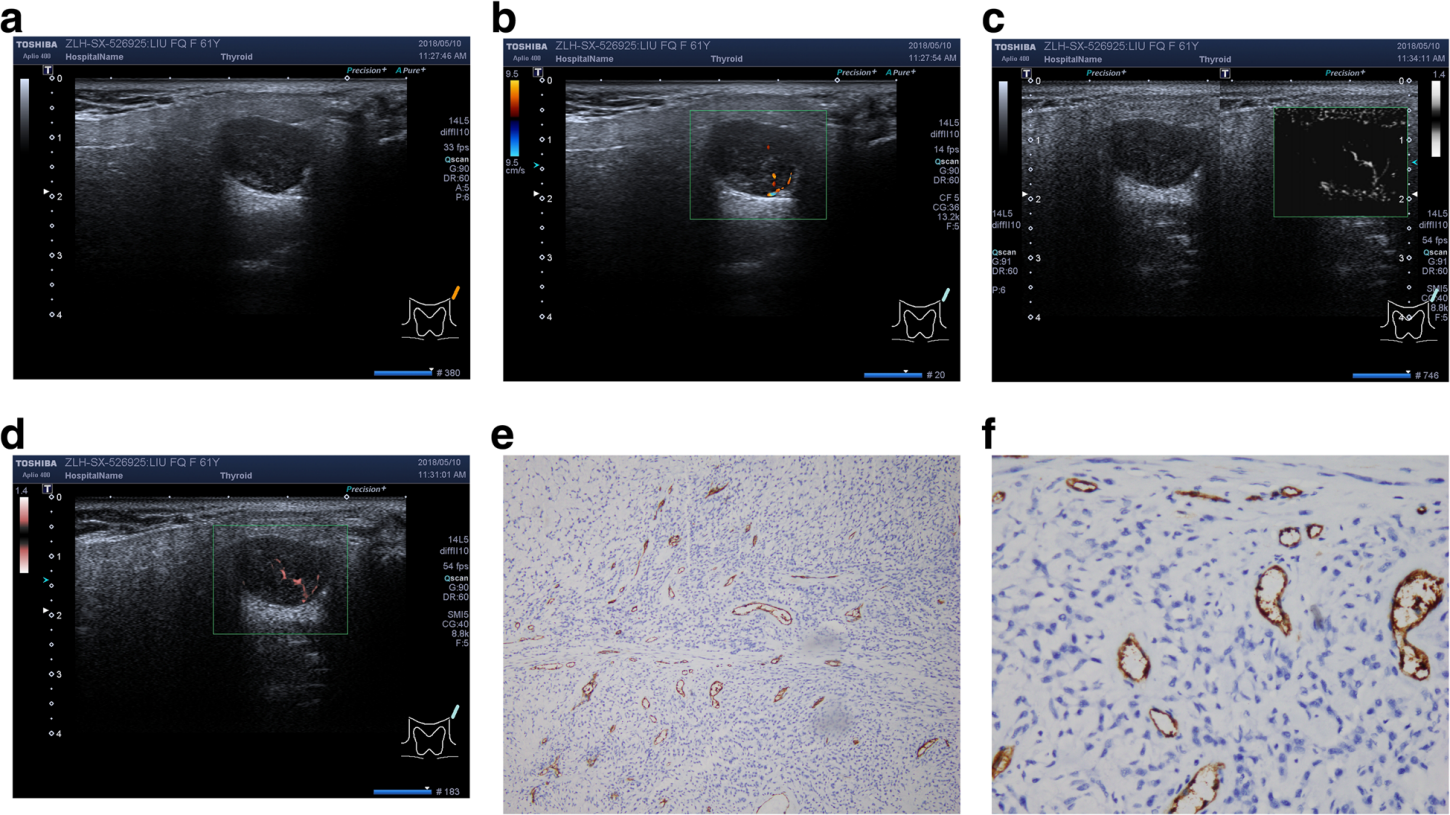
Represent PA case from right parotid gland. **a**. conventional ultrasound image; **b**. CDFI: tiny punctate blood flow, Alder 0;**c**. mSMI and **d**. cSMI: short cosh or rod-like blood flow, central distribution, Alder 2; VI by cSMI: 1.4; **e**. MVD by IHC (× 100); **f**. MVD by IHC: 18 (× 400)

One hundred thirty-six patientshad148 masses of PA (59.4%). In them, 10 patients have ipsilateral multiple tumor, 2 patients have bilateral solitary tumor, the represent images were shown in Fig. 2;

#### BCA group

**Fig. 3:**
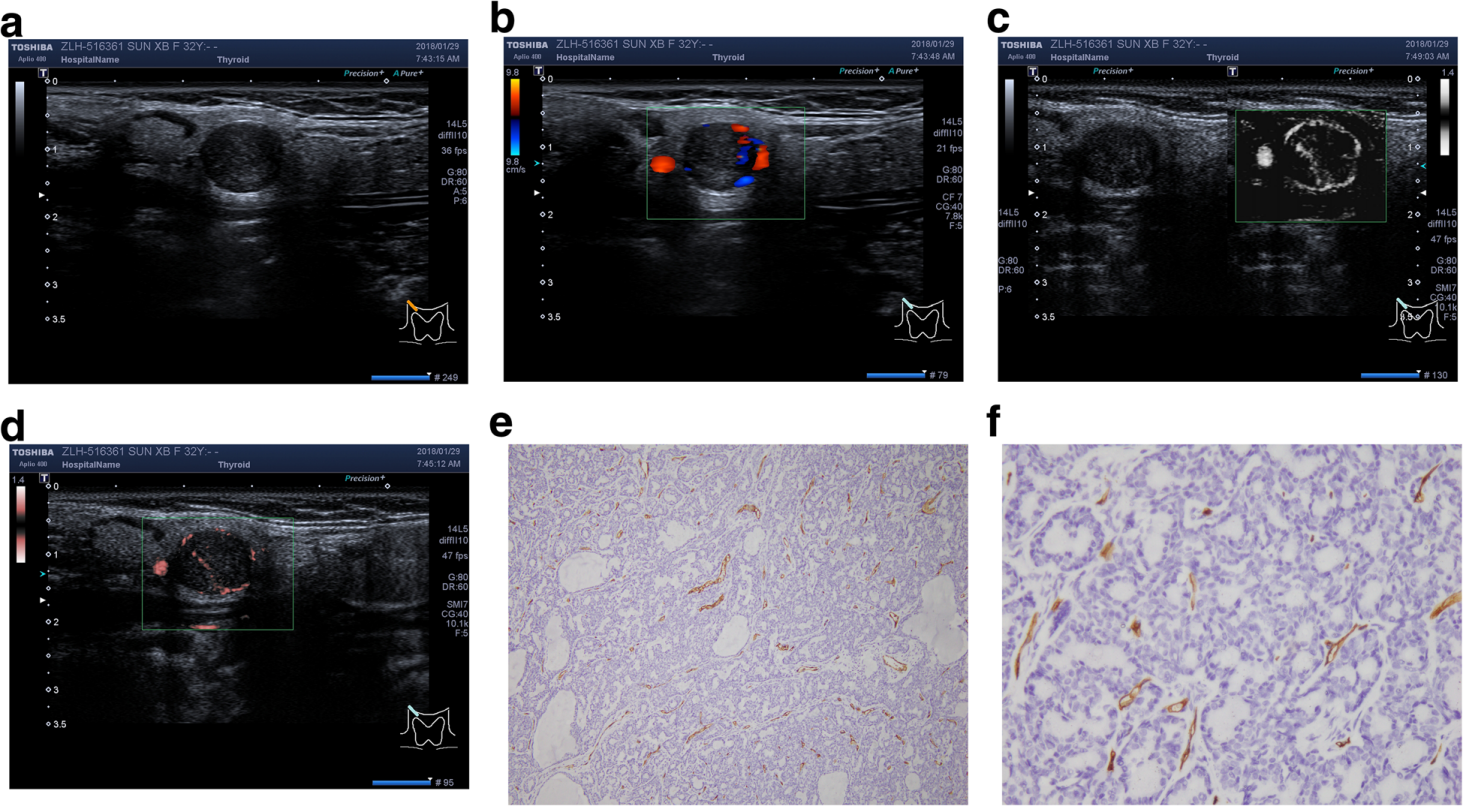
Represent BCA case from left parotid gland. **a**. Conventional ultrasound image; **b**. CDFI: punctate or fine rod vessels, Mixed distribution, Alder 3; **c**. mSMI and **d**. cSMI: dense streak blood stream, Mixed distribution, Alder 3. VIby cSMI:18.1; **e**. MVD by IHC (× 100); **f**. MVD by IHC: 62 (× 400)

Sixteen patients have18 masses of BCA (7.2%). In them, 2 patients have ipsilateral multiple tumors, the represent images were shown in Fig. 3.

#### WT group

**Fig. 4:**
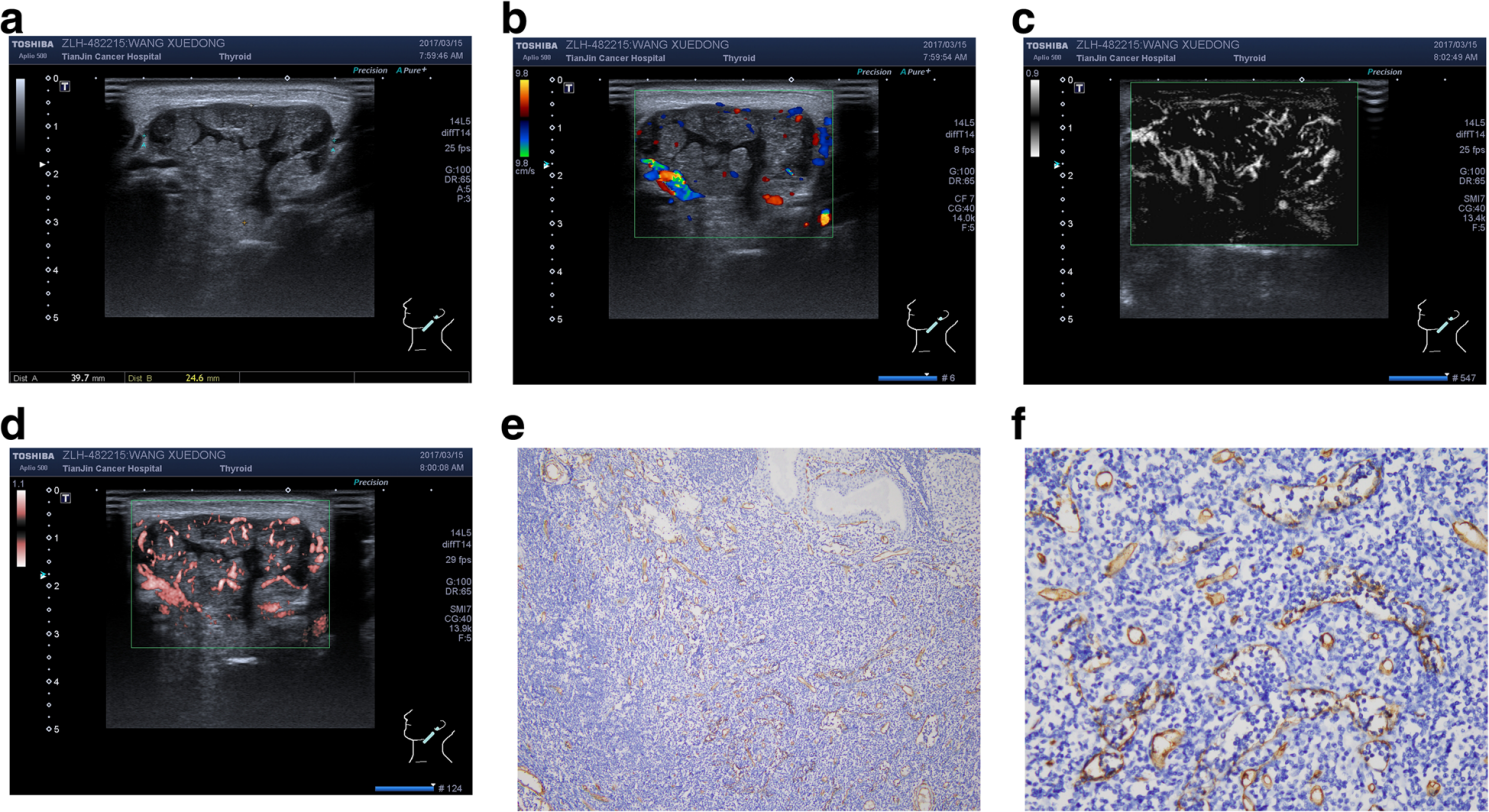
Represent WT case from right parotid gland. **a**. Conventional ultrasound image; **b**. CDFI: 3–4 punctate blood flow, Peripheral distribution, Alder 2; **c**. mSMI and **d**. cSMI: Mixed distribution, Alder 3. VIby cSMI:7.4; **e**. MVD by IHC (× 100); **f**. MVD by IHC: 38 (× 400)

Sixty-five patients had 83 masses of WT (33.3%). In them, 11 patients had ipsilateral multiple tumors, 7 cases have bilateral solitary tumor, the represent images were shown in Fig. 4.

In terms of age and gender, the patients with PA were younger, more common in female, mostly solitary tumor; WT patients are older and more common in male; BCA was older and more common in female. Multiple tumors occurred in all the three groups. Most of the tumors in the three groups were located in the superficial lobe of parotid gland (shown in Table 1).

**Table 1:** General and pathologic character of three types of parotid gland tumors

	PA	BCA	WT	*P* value
Patients *(n)*	136	16	65	
Masses *(n)*	148	18	83	
Solitary/ Multiple *(n)*	124/12	14/2	47/18	0.297*
Age (Mean ± SD)	44.13 ± 15.08	53.19 ± 14.03	58.20 ± 8.23	<0.001**
Sex (*n,* Male/Female)	41/95	4/12	59/6	<0.001*
Location (*n,* Left/Right)	77/71	6/12	48/35	0.164*
Superficial lobe/ Deep lobe *(n)*	137/11	16/2	78/5	0.743*

*:compared by Chi-square and the Fisher’s exact tests; **: compared by one-way ANOVA analysis

### Conventional ultrasound and CDFI of three types of parotid gland tumors

The internal echogenicity of BCA are more homogeneous, while those of PA and WT are more inhomogeneous(*P* < 0.05). There were no statistically significant differences in tumor size, shape, boundary, cystic area, shown in Table 2.

**Table 2:** Conventional ultrasound characters of three types of parotid gland benign tumors

	PA	BCA	WT	*P* value
Size (Maximum diameter, *cm*)	2.71 ± 1.15	2.22 ± 0.79	2.85 ± 0.81	0.07
Shape				
Regular	81	14	55	0.07
Irregular	67	4	28	
Boundary				
Well-defined	134	16	77	0.797
Ill-defined	14	2	6	
Internal echogenicity				
homogeneous	65	11	25	0.02
Inhomogeneous	83	7	58	
Cystic area				
Yes	131	15	63	0.09
No	17	3	20	

There were no statistically significant differences in blood flow distribution and vascular grade of parotid gland masses in three groups by CDFI (shown in Table 3, *P*>0.05).

**Table 3:** Vascular distributions and grade of three types of parotid gland tumors by CDFI

Pathological type	*n*	Vascular Distributions				Vascular Grade			
		Avascular	Peripheral	Central	Mixed	Grade 0	Grade 1	Grade 2	Grade 3
PA	148	47 (31.8%)	45 (30.4%)	32 (21.6%)	24 (16.2%)	47 (31.8%)	46 (31.1%)	30 (20.3%)	25(16.9%)
BCA	18	3 (16.7%)	5 (27.8%)	4 (22.2%)	6 (33.3%)	3 (16.7%)	4 (22.2%)	5 (27.8%)	6 (33.3%)
WT	83	15 (18.1%)	20 (24.1%)	24 (28.9%)	24 (28.9%)	15 (18.1%)	23 (27.7%)	24 (28.9%)	21 (25.3%)
□*χ*^*2*^		11.860				10.071			
*P value*		0.07				0.122			

### SMI of three types of parotid gland tumors

The differences in vascular distribution and vascular grade of parotid gland masses in the three groups via SMI technique were statistically significant (*P* < 0.05). The vascular distribution and vascular grade in PA were mainly peripheral (33.1%) and avascular (25.7%), and vascular Grade 1 (27.8%) and Grade 0 (25.7%). In WT, central (31.3%) and mixed distribution (34.9%) were common, and Grade 3 (37.3%) and Grade 2 (36.2%) were dominant. BCA was mainly peripheral (33.3%) and mixed distribution (33.3%), and in Grade 2 (33.3%) and Grade 3 (33.3%). (all *P* < 0.05, Table 4).

**Table 4:** Vascular distributions and grade of three types of parotid gland tumors by SMI

Pathological type	*n*	Vascular Distributions				Vascular Grade				VI
		Avascular	Peripheral	Central	Mixed	Grade 0	Grade 1	Grade 2	Grade 3	
PA	148	38 (25.7%)	49 (33.1%)	29 (19.6%)	32 (21.6%)	38 (25.7%)	41 (27.8%)	37 (22.9%)	32 (21.6%)	4.57 ± 2.40
BCA	18	2 (11.1%)	6 (33.3%)	4 (22.3%)	6 (33.3%)	2 (11.1%)	4 (22.3%)	6 (33.3%)	6 (33.3%)	6.89 ± 3.47
WT	83	8 (9.6%)	20 (24.1%)	26 (31.3%)	29 (34.9%)	7 (9.6%)	15 (16.9%)	29 (36.2%)	32 (37.3%)	8.89 ± 3.87
□*χ*^*2*^		16.175				20.413				44.012
*P value*		0.013				0.002				<0.001

With SMI, 19.3% of parotid benign tumors was avascular, and the detection rate of blood flow was 81.1%. Detection rate of blood vessels by CDFI was 73.9%. The detection rate of blood vessels of SMI was higher than that of CDFI. The overall detection rate of SMI for Alder 2 and 3 was significantly higher than that of CDFI (57.0% vs.44.6%). (*P* < 0.05, Table 5).

**Table 5:** Comparing the proportion of different vascular distributions and grade for parotid gland tumors by CDFI and SMI

	Vascular Distributions				Vascular Grade			
	Avascular	Peripheral	Central	Mixed	Grade 0	Grade 1	Grade 2	Grade 3
CDFI	65 (26.1%)	70 (28.1%)	60 (24.1%)	54 (21.7%)	65 (26.1%)	73 (29.3%)	59 (23.7%)	52 (20.9%)
SMI	47 (18.9%)	75 (30.1%)	59 (23.7%)	67 (26.9%)	47 (18.9%)	60 (24.1%)	72 (28.9%)	70 (28.1%)
χ2	4.468				8.109			
*P* value	0.215				0.040			

VI were lowest in PA, highest in WT, BCA is in moderate, the differences were statistically significant. (*P* < 0.001, Table 6).

**Table 6:** Comparing the mean difference of VI and MVD of three types of parotid gland tumors

Pathological type	PA	BCA	WT	*F*	*P* value
VI	4.57 ± 2.40	6.89 ± 3.47	8.89 ± 3.87	44.012	<0.001
MVD	29.31 ± 15.11	37.94 ± 12.45	48.33 ± 15.64	42.177	<0.001

### Correlation analysis of vascular index (VI) and microvascular density (MVD)

**Fig. 5:**
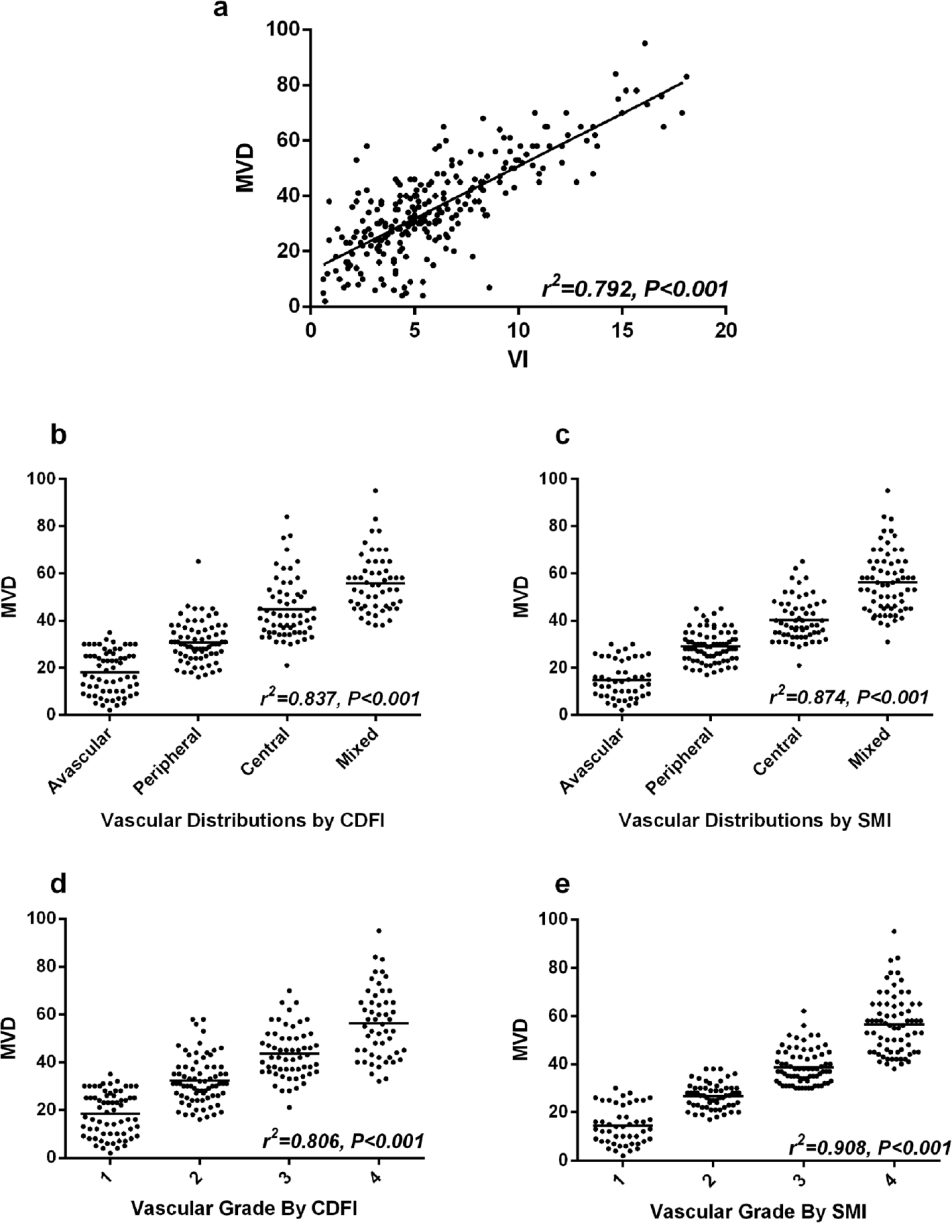
The correlation analysis of MVD and VI. **a**. The correlation analysis of MVD and VI by cSMI; **b**. The correlation analysis of MVD and vascular distributions by CDFI; **c**: The correlation analysis of MVD and vascular distributions by SMI; The correlation analysis of MVD and vascular grade by CDFI; **e**. The correlation analysis of MVD and vascular grade by SMI

VI were in line with MVD, MVD was also lowest in PA, highest in WT, BCA is moderate, the differences were statistically significant (*P* < 0.001, Table 6). The MVD was correlated with VI by Pearson correlation analysis(*r*^*2*^ = 0.792, *P* < 0.001, Fig. 5a).

In further, the vascular distribution and grade by SMI and CDFI were also correlated with MVD by Spearman correlation analysis, shown in Fig. 5b-e. Yet, the correlation index between vascular distribution and grade by SMI(*r*^*2*^ = 0.874, *P* < 0.001; *r*^*2*^ = 0.908, *P* < 0.001) and MVD were significantly higher than that by CDFI(*r*^*2*^ = 0.837, *P* < 0.001, *r*^*2*^ = 0.806, *P* < 0.001).

## Discussion

High-frequency ultrasound is a first-line examination method for the diagnosis of parotid gland tumor, which can provide information such as the location, size, shape, internal echo and the relationship with surrounding tissues, etc. However, due to the diversity of parotid gland tumor pathological types, ultrasound features overlap greatly, common ultrasound has high sensitivity but low specificity for differential diagnosis. In this study, there were no statistically significant differences in conventional ultrasound characters including tumor boundary, shape, cystic area and size between these three different parotid gland tumors. Generally, parotid gland tumor is small, with clear boundaries and regular shapes. They are round or oval, with complete capsule. Some of the parotid gland tumor have complex characters, which are lobulated and irregular in growth. Cystic changes may occur in larger tumors and in all three types of tumors, which is results from liquefaction necrosis, internal bleeding, or mucous retention secreted by tumor epithelium.

In this study, there were statistically significant differences in internal echogenicity among the three groups of parotid gland tumors. The internal echogenicity of PA and WT were more inhomogeneous. PA is a borderline tumor, with atypia, composed of myxoid epithelial tissue and (or) chondroid tissue. The internal echogenicity depends on tumor tissue, epithelia dominated tumors are usually hypoecho, mucinous or cartilaginous dominated tumors are usually extreme hypo-echo. WT is composed of epithelial tissues and lymphoid tissues, the echoes of lymphoid tissues are hypoecho, while the epithelial tissues of the tumor are functional and secrete some liquid, so the cystic area in the tumor are common, the internal echogenicity inside the tumor extremely-hypo, and some of them have “grid-like”. BCA are composed of a single cell, which constitutes the basal cell layer. The layers are relatively sole, without interstitial and cartilaginous matrix, and the internal echoes are more homogeneous. Echogenicity inside the tumor is helpful to distinguish BCA from PA and WT.

Ultrasonic Doppler signals are not only derived from the blood flow, but also from the motion artifacts (clutter) of tissues. The motion artifacts have high amplitude and low velocity, and are often mixed with the low-velocity blood flow signals, or even conceal the micro blood flow signals. The CDFI technology can’t distinguish motion artifact and blood flow signal, because its processing method is a one-dimensional filtering technology. Low-speed motion artifacts and low-speed blood flow information were filtered indiscriminately. Therefore, CDFI could only display vessels with diameter of > 0.2 mm and blood flow signals with relatively high flow velocity, and could only provide the general blood supply situation in the tumor [15]. CDFI lacks the ability to evaluate the small blood vessels in the tumor, and could not reflect the real microvascular situation in the tumor. The growth of solid tumors is vascular dependent. As an important component of tumor stroma, blood vessels interact with tumor cells to form a complete microenvironment. This study showed that the detection rate of SMI for benign parotid tumor blood vessels was significantly different from CDFI, and the overall detection rate of SMI for Grade 2 and 3 was significantly higher than CDFI. In clinic, SMI technology use adaptive algorithm to distinguish motion artifacts and slow blood flow, fully suppress noise using the unique processing technology, highlight slow blood flow signals intelligence, brings high sensitivity to subtle low speed blood flow. So it can clear display diameter > 0.1 mm microvascular blood flow signal at low speed, show the tiny blood vessels without using the contrast agent and achieve the similar imaging display effect. Thus, SMI can more truly present the microvascular condition in the tumor than CDFI [15].

The number of micro vessels inside the tumor is closely related to the pathological type of the tumor. Usually the blood supply of the benign tumor is less, if the blood supply of the benign tumor increases, it indicates the possibility of malignant transformation. In this study, CDFI showed more red and blue blood flow distribution in the interior and peripheral in WT, SMI showed more puncture vessels in WT, more dendritic blood flow, mainly central or mixed distribution, 73.5% of patients with Grade 2–3 blood supply, significantly more than other benign tumors in parotid gland. This is related to its pathologic structure. WT is composed by both epithelial tissue and lymphoid tissue, its epithelial components form irregular large glandular ducts or microcysts, and form nipple into cavity, hyperplasia of lymphoid tissue forming lymphoid follicles. Most of the WT mesenchymal percentage was near to epithelial, and micro vascularity was rich in lymphoid interstitial [16]. The higher the content of mesenchymal, the more abundant the distribution of micro vessels inside and outside the interstitial and capsule, which makes WT significantly different from other benign tumors in parotid gland in terms of blood supply. Because the lesion originates from the heterotopic glandular epithelial cells in the lymph node, the blood flow distribution is close to the lymphatic portal blood flow, so the blood flow is usually dendritic. PA is often circumscribed by the edge of blood flow, and the blood flow signals are not rich, mainly with no blood flow and peripheral blood flow, Grade 0 and Grade 1. Some PA blood flow signals are rich, even up to Grade 3. The reason is related to local tumor capsule infiltration, or focal growth to the capsule. Most blood flow of BCA are also rich, mainly peripheral and mixed distribution, Grade 2 and Grade 3, which is related to the pathological characteristics of the tumor. Pathologically, BCA has more characteristic vascular network arranged along the endothelium, mainly capillaries and venules, and blood flow is mostly peripheral distribution.

The occurrence and development of solid tumors need two stages: the perivascular phase stage and the vascular stage. The diameter of tumor in pre-vascular stage was < 2 mm, and the tumor grew slowly, mainly relying on osmosis to obtain nutrition. When the tumor continues to grow to > 2 mm in diameter, it enters the vascular stage, with a large number of micro angiogenesis, which mainly relies on angiogenesis to obtain nutrition [17]. Pathological MVD is the gold standard for evaluating tumor microvascular condition. At present, the main imaging methods for evaluating tumor micro vessels include CEU and enhanced MRI, which are highly correlated with MVD [18, 19]. However, there is no noninvasive imaging technique to evaluate the angiogenesis of parotid gland tumors in real time. Therefore, it is urgent to find an imaging method with good correlation with MVD and no trauma, which can evaluate tumor angiogenesis in the preoperative living state and provide valuable prognostic information for clinical use. SMI quantitative index VI can accurately detect the pixel ratio of blood flow signal in parotid gland tumor, and truly reflect the distribution density of low-velocity blood flow signal in the tumor, so as to intuitively reflect the microvascular condition of the tumor. In this study, VI value and MVD value of PA, BCA and WT were successively increased, and VI was significantly positively correlated with MVD, implied that VI could accurately and quantitatively reflect the micro-blood flow generation in parotid gland tumor.

## Conclusion

Despite the parotid benign neoplasm in conventional ultrasonic features and CDFI have overlap, but the SMI evaluation can provides us with abundant diagnostic information on flow classification, distribution and blood flow in VI, to assist conventional ultrasound, the SMI can significantly improve the ability of ultrasonography in the differential diagnosis of parotid benign tumor, provide reference for clinical diagnosis and treatment decisions. In summary, SMI can provide low-velocity blood flow information that can’t be displayed by other blood flow imaging technologies, which is helpful for the differential diagnosis of common benign tumors of parotid gland, and is expected to be more widely used.

## Data Availability

The datasets used during the current study are available from the corresponding author on reasonable request.

## Abbreviations

SMI: Superb microvascular imaging
PA: Pleomorphic adenoma
WT: Warthin’s tumor
BCA: Basal cell adenoma
CEU: Contrast-enhanced ultrasound
CDFI: Color doppler flow imaging
IHC: Immunohistochemical
ROI: Region of interest
VI: Vascular index
mSMI: SMI image includes grayscale mode
color, cSMI: Color mode
MVD: Micro vessel density (MVD)
